# The IGF/Insulin-IGFBP Axis in Corneal Development, Wound Healing, and Disease

**DOI:** 10.3389/fendo.2020.00024

**Published:** 2020-03-03

**Authors:** Whitney L. Stuard, Rossella Titone, Danielle M. Robertson

**Affiliations:** Department of Ophthalmology, UT Southwestern Medical Center, Dallas, TX, United States

**Keywords:** cornea, IGF-1, IGF-1R, INSR, Hybrid-R, IGFBP-2, IGFBP-3

## Abstract

The insulin-like growth factor (IGF) family plays key roles in growth and development. In the cornea, IGF family members have been implicated in proliferation, differentiation, and migration, critical events that maintain a smooth refracting surface that is essential for vision. The IGF family is composed of multiple ligands, receptors, and ligand binding proteins. Expression of IGF type 1 receptor (IGF-1R), IGF type 2 receptor (IGF-2R), and insulin receptor (INSR) in the cornea has been well characterized, including the presence of the IGF-1R and INSR hybrid (Hybrid-R) in the corneal epithelium. Recent data also indicates that each of these receptors display unique intracellular localization. Thus, in addition to canonical ligand binding at the plasma membrane and the initiation of downstream signaling cascades, IGF-1R, INSR, and Hybrid-R also function to regulate mitochondrial stability and nuclear gene expression. IGF-1 and IGF-2, two of three principal ligands, are polypeptide growth factors that function in all cellular layers of the cornea. Unlike IGF-1 and IGF-2, the hormone insulin plays a unique role in the cornea, different from many other tissues in the body. In the corneal epithelium, insulin is not required for glucose uptake, due to constitutive activation of the glucose transporter, GLUT1. However, insulin is needed for the regulation of metabolism, circadian rhythm, autophagy, proliferation, and migration after wounding. There is conflicting evidence regarding expression of the six IGF-binding proteins (IGFBPs), which function primarily to sequester IGF ligands. Within the cornea, IGFBP-2 and IGFBP-3 have identified roles in tissue homeostasis. While IGFBP-3 regulates growth control and intracellular receptor localization in the corneal epithelium, both IGFBP-2 and IGFBP-3 function in corneal fibroblast differentiation and myofibroblast proliferation, key events in stromal wound healing. IGFBP-2 has also been linked to cellular overgrowth in pterygium. There is a clear role for IGF family members in regulating tissue homeostasis in the cornea. This review summarizes what is known regarding the function of IGF and related proteins in corneal development, during wound healing, and in the pathophysiology of disease. Finally, we highlight key areas of research that are in need of future study.

## Introduction

**Figure 1:**
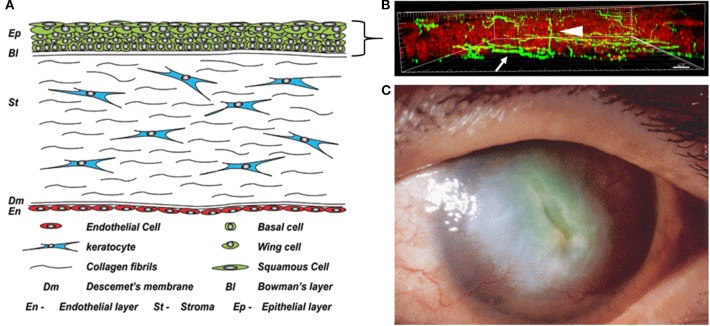
The cornea in health and disease. **(A)** Anatomical schematic showing all five cell layers in the cornea. A five to seven stratified layer of epithelial cells (basal, wing, and squamous) composes the corneal epithelium. Keratocytes, normally quiescent cells, reside in the corneal stroma which consists of intertwining collagen fibrils. Finally, there is a single endothelial cell layer on the innermost layer of the cornea that faces the interior of the eye and is responsible for maintaining stromal hydration. Figure taken from Polisetti et al. (1). **(B)** Maximum intensity projection of nerves labeled with neuronal beta tubulin (green) in the mouse cornea *in situ*. Epithelial nuclei are counterstained with propidium iodide in red. Nerve fibers that run among the basal layer of the epithelium and just beneath it form the subbasal nerve plexus (arrow). Intra-terminal nerve fibers branch perpendicularly from the subbasal nerve plexus and run throughout the corneal epithelium to the surface of the eye (arrowhead). Figure taken from Cai et al. (2). **(C)** A cornea with diabetic keratopathy. Note the large central opacification and significant neovascularization. Image taken from Matsumoto et al. (3).

The cornea constitutes the outer covering of the eye and provides two thirds of the refractive power necessary for vision. It is composed of five layers including a stratified epithelium, Bowman's membrane, the collagenous stroma, Descemet's membrane, and the single cell layered endothelium (Figure 1A). Corneal innervation is supplied by the ophthalmic branch of the trigeminal nerve. While corneal innervation is described in detail elsewhere and is beyond the scope of this review, it is important to note that the corneal epithelium is the highest innervated structure in the body due to the high density of intraepithelial nerve terminals (Figure 1B) (4). This, along with the tight barrier function of the epithelium, plays a major role in protecting intraocular structures from the outside environment. In addition to protection, the cornea must maintain a smooth, transparent, and avascular appearance to allow for the passage of light. The avascular nature of the cornea is one of the cornea's many unique properties. Other important properties include the peripheral location of stem cells in the corneal limbus, the paired movement of daughter cells in the central cornea from the basal layer to the surface epithelium, the epithelium's exceedingly high glycogen content, and the precise organization of collagen lamellae that facilitates transparency.

Corneal wound healing is complex and requires a unique orchestration of events including resurfacing of the corneal epithelium, deposition of basement membrane, and regeneration of the extracellular matrix. At the level of the epithelium, immediately upon wounding, the basement membrane is disassembled and epithelial cells surrounding the wound margin migrate as a sheet to cover the wound. In the presence of an incisional wound, epithelial cells migrate down into the wounded stroma. Once the wounded area is fully covered by these flattened epithelial cells, proliferation of limbal and transient amplifying cells ensues, followed by migration, stratification, the re-establishment of junctional proteins, and the restoration of the basement membrane. When wounds extend beyond the epithelium into the corneal stroma, cytokines released by epithelial cells and present in tears transform the normally quiescent keratocytes into fibroblasts that migrate into the wounded area. This is followed by their sequential transformation into myofibroblasts, strong contractile cells that function to close the wound. Ultimately, over time, keratocytes repopulate, and remodel the extracellular matrix. However, abnormal extracellular matrix often persists, resulting in corneal fibrosis. Unlike the corneal epithelium, when damaged, the corneal endothelium does not proliferate to close a wound. Instead, endothelial cells flatten and spread, taking on a polymorphic appearance.

Many systemic diseases, such as diabetes, can dramatically alter the normal biology of the cornea, resulting in a thinned, dysplastic epithelium with damaged corneal nerves, and cell loss in the endothelial monolayer, the latter of which drives corneal swelling. Most notably, since diabetes can negatively impact all layers of the cornea, impaired corneal wound healing can present a major clinical problem that is often refractory to therapy. While greatly under-recognized, corneal complications occur in 40–70% of diabetics (5). These complications, which range from mild to severe, can result in chronic and painful corneal complications, predispose the cornea to infection, and in advanced stages, lead to neurotrophic disease and blindness (Figure 1C) (6, 7). The importance of proper growth factor signaling in the normal and diabetic cornea is well-established. The focus of this review is to chronicle what is known about the localization and function of insulin-like growth factor (IGF) family members in the cornea and to highlight critical areas of investigation for future studies.

## The IGF System

**Figure 2:**
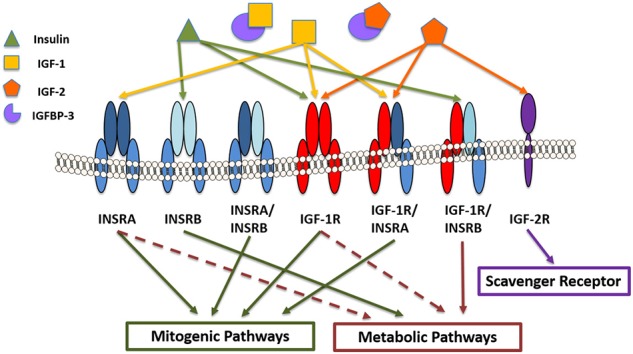
IGF/insulin-IGFBP family members in the corneal epithelium. Receptors include insulin receptor A and B (INSRA and INSRB), IGF type 1 and type 2 receptors (IGF1-R and IGF-2R) and the hybrid receptors (INSRA/INSRB, IGF-1R/INSRA, and IGF1-R/INSRB). Insulin binds with higher affinity to INSRB and the hybrid receptor (IGF-1R/INSRB), predominantly activating metabolic pathways. IGF-1 and IGF-2 bind with higher affinity to IGF-1R and IGF-1R/INSRA, activating mitogenic signaling. IGF-2 also binds the scavenger receptor, IGF-2R. IGF binding protein-3 (IGFBP-3) sequesters IGF-1 and IGF-2, preventing them from binding to their cognate receptors.

The insulin-like growth factor (IGF) system consists of two peptide ligands, IGF-1 and IGF-2, and the hormone insulin (Figure 2). These extracellular ligands activate the IGF Type 1 receptor (IGF-1R), the IGF type 2 receptor (IGF-2), and insulin receptor (INSR), all with varying affinities. The system is further regulated at the extracellular level by the presence of IGF-binding proteins (IGFBPs). There are six known IGFBPs. Historically, IGFBPs function to bind IGF-1 to prolong its half-life in circulation and to prevent IGF-1 induced activation of IGF-1R. This is mediated by proteolytic enzymes that function to cleave IGFBPs, thereby regulating the amount of bioavailable IGF-1. To date, two known proteases have been identified. They include the pregnancy-associated plasma proteins, PAPP-A, and PAPP-A2, which are inhibited by the stanniocalcins (8, 9). In addition, the ligands, as well as binding proteins, have been shown to interact with IGF family receptors to exert unique effects that are cell and tissue dependent (10, 11). IGF-1 is well-known for its role in growth and development in physiologically healthy tissues (12). In the cornea, IGF-1R, IGF-2R, and INSR and their canonical ligands have been studied in the epithelium, stroma, and endothelium. As seen in other tissues, these works have demonstrated key biological roles for members of the IGF family in proliferation, homeostasis, differentiation, and wound healing.

## Characterization of IGF-1R, IGF-2R, and INSR in the Human Cornea

Structurally, IGF-1R and INSR are transmembrane tetrameric glycoproteins comprised of two transmembrane beta subunits, each containing an intracellular tyrosine kinase domain, and two alpha subunits, each containing an extracellular ligand binding domain (13). In contrast to these receptors, IGF-2R is a monomeric transmembrane protein with 15 different extracellular domains (14). INSR differs from IGF-1R and IGF-2R in that it undergoes alternative splicing at exon 11 resulting in two different isoforms, INSRA and INSRB. Each isoform is thought to play distinct roles in development and metabolism. Expression of each also mediates affinity for insulin, kinase activity, and may contribute to the rate of internalization and receptor recycling.

INSR was first identified in the cornea by Naeser in the late 90's (15). In that work, he used immunohistochemical techniques to stain for INSR in donor human corneas with and without diabetic retinopathy. He found strong staining for INSR in the corneal epithelium, stromal keratocytes, and endothelium. Staining was unchanged in diabetes. Rocha and colleagues also used immunohistochemical techniques to show that INSR was indeed expressed in the corneal epithelium (16). In their study, INSR localized to the cytoplasm and plasma membrane primarily in the wing and superficial cell layers. Varied expression was evident in the basal and intermediate suprabasal cell layers. While not reported, INSR appeared to be expressed in nuclei of corneal epithelial cells. More recent work by our laboratory has used multiple complementary approaches to confirm expression of INSR in corneal epithelial cells and to define the intracellular localization of this protein (as discussed in greater detail later in this manuscript).

Nakamura first used ligand-binding assays to confirm the presence of IGF-1R in corneal epithelial cells and showed that IGF-1R had the greatest affinity for IGF-1, followed by IGF-2, and then insulin (17). As they did for INSR, Rocha also stained for the presence of IGF-1R in the corneal epithelium. Using an antibody that recognizes the extracellular alpha subunit of IGF-1R, they demonstrated robust staining at the plasma membrane throughout all epithelial layers. Subsequent studies by our laboratory have further characterized the expression and localization of IGF-1R in cultured corneal epithelia (11, 18, 19). Using antibodies that recognize both the alpha and beta subunits of IGF-1R, we found that the full mature receptor localized to the nucleus of corneal epithelial cells. We confirmed this using subcellular fractionation and immunoblotting assays. We further found that IGF-1R interacts with E-cadherin at areas of cell-cell junctions (18). Interactions between IGF-1R and E-cadherin have been previously reported in tumor biology where they are thought to regulate tumor invasion and in Madin Darby canine kidney cells (20, 21). In this latter cell type, is was shown that binding to E-cadherin negatively regulated ligand-induced activation of IGF-1R (20). The significance of the interaction between IGF-1R and E-cadherin in the corneal epithelium is unknown. Consistent with a growth inhibitory role, we postulate that E-cadherin binding is necessary to attenuate IGF-1R activation and downstream signaling events that mediate proliferation and growth (18).

It has long been known that IGF-1R and INSR are highly homologous receptors, with 45–65% homology in the amino acid sequence in the alpha subunit and 84% homology in the beta subunit (22). Due to this high level of homology, IGF-1R and INSR can hybridize to form an IGF-1R/INSR hybrid (Hybrid-R). Hybrid-Rs can form with either INSR isoform. Hybrid-R is expressed in the corneal epithelium (19). Using dithiothreitol to cleave the class 1 disulfide bonds and separate IGF-1R alpha/beta subunits from INSR alpha/beta subunits following stimulation with IGF-1 or insulin, we confirmed that Hybrid-R is activated by IGF-1 and not insulin in corneal epithelial cells. Since INSR isoform A is predominantly activated by IGF-1 to promote IGF-1 signaling, whereas INSR isoform B is predominantly activated by insulin and functions to attenuate IGF-1 signaling, our data suggest that in the corneal epithelium, Hybrid-R is composed of IGF-1R and INSR isoform A (19). This finding remains to be confirmed at a protein level.

Bohnsack and colleagues evaluated IGF-2R distribution in human, murine, and porcine corneas. In their study, they found that IGF-2R was present throughout all cell layers of the cornea (23). In murine and porcine models, expression was primarily localized to the basal epithelium. After wounding, they reported an 11-fold increase in IGF-2R in the stroma and epithelium. The increase in IGF-2R was associated with an increase in differentiation of fibroblasts to myofibroblasts, demonstrated by an increase in α-SMA expression, which was subsequently blocked by shRNA knockdown of IGF-2R. Likewise, when keratocytes were cultured in serum free media and treated with TGF-beta to induce myofibroblast differentiation *in vitro*, there was a similar increase in IGF-2R expression in myofibroblasts, at the mRNA and protein level. Together, these findings suggest a potential role for IGF-2R in mediating fibroblast to myofibroblast transformation during wound healing.

## Intracellular IGF-1R, INSR, and HYBRID-R

More recently, non-canonical roles for IGF-1R and INSR have been suggested (24). In their seminal paper, Sehat et al. used human melanoma (DFB) and leiomysocarcoma (SKUT-1) cells along with human embryonic kidney cells (HEK 293) to first describe the nuclear localization of IGF-1R (25). Using a serum-based model, they demonstrated that serum starvation depletes IGF-1R from the nucleus and that treatment with IGF-1 induced translocation of IGF-1R from the plasma membrane to the nucleus. They further showed that translocation to the nucleus was mediated by SUMOylation of IGF-1R by the SUMO modifier SUMO-1. It has since been shown that nuclear IGF-1R interacts with several different nuclear proteins and functions to regulate the cell cycle, DNA damage responses, invasion, and metastasis (26–29).

Like IGF-1R, studies have also reported that INSR localizes to the nucleus. In the late 70's, Goldfine and colleagues were the first to demonstrate that insulin bound to isolated nuclei *in vitro* (30). They further showed that treatment of isolated nuclei with trypsin prevented insulin binding. The authors concluded that a hormone receptor modulated by insulin was present in rat liver nuclei. Just over a decade later, a second group refuted these earlier findings. In their study, Soler et al. used a combination of *in vitro* techniques to investigate a potential nuclear localized receptor (31). In contrast to the prior work, they concluded that once dissociated from INSR, the biologically intact and active hormone accumulated in the nucleus and associated with heterochromatin. Until recently, few studies have followed up on the potential for a nuclear-localized INSR. In rat hepatocytes, INSR has been shown to translocate to the nucleus where it regulates calcium signals and proliferation (32). Hancock and colleagues have also investigated the role of INSR in the nucleus of mouse liver cells (33). In their studies, they found that INSR directly associated with genome-wide promoters and regulates gene expression through interactions with RNA polymerase II.

Consistent with these studies, we have found that both IGF-1R and INSR localize to the nucleus of corneal epithelial cells (18). Unlike prior studies however, we have found that the nuclear localized receptor is Hybrid-R (19). We have further shown that Hybrid-R nuclear translocation occurs in response to growth factor withdrawal and is not induced by stimulation with IGF-1. Instead, expression and localization of each receptor is mediated by insulin (11). In the absence of insulin, expression of IGF-1R and INSR is upregulated and the receptors accumulate as Hybrid-R in the corneal epithelial cell nucleus. This is mediated through SUMOylation by the SUMO modifier SUMO2/3. The ability of insulin levels to regulate Hybrid-R nuclear translocation is due to its ability to regulate extracellular levels of IGFBP-3. Studies in our laboratory which decrease expression of IGFPB-3 using siRNA knockdown followed by the addition of exogenous recombinant human IGFBP-3 not only demonstrate robust translocation to the nucleus, but also drive receptor accumulation in the insoluble nuclear fraction, indicating association with DNA (11). While we have been unable to detect the presence of IGF-1R alone in the nucleus, we have not yet ruled out the presence of INSR not complexed with IGF-1R.

Our more recent studies on the function of intracellular IGF-1R, INSR, and Hybrid-R have led to the novel observation that INSR and IGF-1R are present in mitochondria (34). Using mitochondrial fractionation assays, we have confirmed that IGF-1R and INSR localize to mitochondria and that expression of both accumulates during stress induced by growth factor withdrawal. Using reciprocal immunoprecipitation, we have further found that INSR and IGF-1R bind the voltage gated anion channel, VDAC1. Importantly, when we disrupt the interaction between INSR and VDAC1 using INSR knockdown, we see robust mitochondrial ring/donut shaped fragmentation. This finding indicates that the novel interaction between INSR and VDAC1 is important for mediating mitochondrial stability (34).

## Insulin and Glucose Uptake

**Figure 3:**
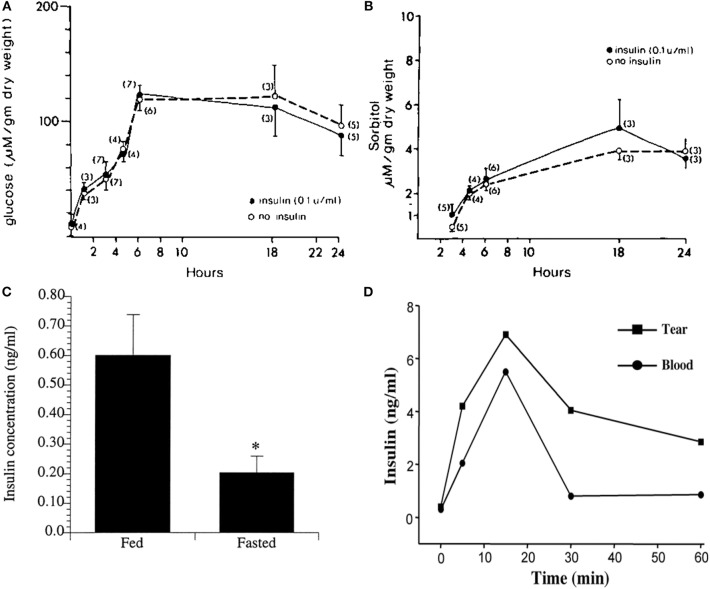
Corneal epithelial cells are “insulin insensitive” and do not require insulin for glucose uptake. **(A)** Glucose content (micromoles per gram ± SEM) of corneal epithelial cells after incubation with 35 mM glucose in TC-199 with and without insulin. **(B)** Corneal epithelial cell sorbitol (micromoles per gram ± SEM) content after incubation with 35 mM glucose in TC-199 with and without insulin. A&B adapted from Friend et al. (39). **(C)** Radioimmunoassay (RIA) measurement of mean insulin levels present in tears of fasted vs. fed human subjects, *P* < 0.05. Adapted from Rocha et al. (16). ^*^P < 0.05. **(D)** Insulin levels in tears measured by RIA over time after systemic administration of glucose (1 g/kg body wt) in rats. Adapted from Cunha et al. (40).

Insulin is a peptide hormone that functions to mediate metabolic effects in addition to growth and proliferation. Structurally, bioactive insulin presents in humans as a monomer consisting of two chains: an A-chain and B-chain, joined by disulfide bonds (35, 36). Insulin is produced by beta cells in the pancreas, where it is stored and secreted in response to high levels of blood glucose (37). In most cell types and tissues, insulin is required for glucose uptake. In tissues such as skeletal muscle, adipose tissue, and lungs, insulin binds INSR and promotes the uptake of glucose from circulation through glucose transporter-4 (GLUT4) (38). In certain tissues however, such as the corneal epithelium, glucose uptake is insulin independent, meaning that corneal epithelial cells do not require insulin for glucose uptake (Figures 3A,B) (39, 40). Instead, glucose uptake is mediated by a constitutively active glucose transporter, GLUT1 (41). This allows for the continuous passage of glucose into cells (39). In conditions where the metabolic demand is increased, such as following a wound, the corneal epithelium responds by increasing the number of GLUT1 transporters in order to provide sufficient energy for proliferation, migration, and survival (42).

Using a rat corneal wound model, Takahashi and colleagues found that levels of both GLUT1 mRNA and protein were increased in the corneal epithelium as early as 4 h after wounding, peaking at 2 days post-injury (43). They hypothesized that the increase in GLUT1 was necessary to facilitate increased glucose uptake and provide fuel to promote wound healing. In a subsequent study by the same group, they used streptozotocin, a drug known to kill insulin-producing pancreatic beta cells, to induce Type 1 diabetes mellitus. They then examined GLUT1 expression before and after wound healing. They found that in response to the wound, GLUT1 was similarly increased in both diabetic and control groups compared to the non-wounded controls. However, there was no difference in receptor expression prior to wounding between diabetic and non-diabetic animals. Together, their findings indicated that GLUT1 expression had no impact on delayed corneal wound healing in diabetes. This work is in agreement with early studies done by Kumagai et al. that also failed to show any differences in GLUT1 expression in the diabetic vs. non-diabetic human cornea (44).

In addition to its role in glucose homeostasis, insulin has broader cellular functions including regulation of cell metabolism, autophagy, apoptosis, growth, and proliferation (45). While insulin is not required for glucose uptake, insulin and its receptors are present in the human cornea and tear film (16). Moreover, insulin levels are increased in tears in fed individuals compared to fasted (Figure 3C). Similar to humans, insulin is increased in the rat tear film following a single bolus of glucose administered intravenously (Figure 3D) (40). It is unknown whether tear derived insulin is taken up by terminally differentiated surface epithelial cells or is able to somehow cross the tight epithelial barrier, the latter of which is unlikely. Thus, the functional significance of insulin in tear fluid is unknown.

## Insulin and Metabolism in Corneal Epithelial Cells

**Figure 4:**
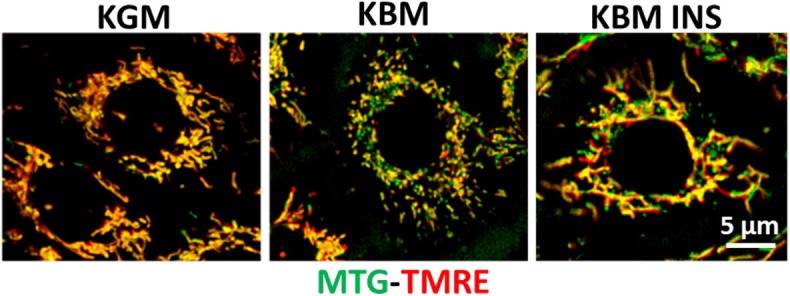
Insulin blocks loss of mitochondrial depolarization. hTCEpi cells were cultured for 48 h in KGM or KBM with or without 5 μg/ml of insulin. Mitochondria were stained with MitoTracker green (MTG, green), a marker of mitochondrial morphology, and TMRE (red), a marker for polarized mitochondria. Culture in basal media without growth factor supplements resulted in mitochondrial depolarization and fragmentation. Concurrent treatment with insulin blocked depolarization and stimulated mitochondrial elongation. KGM, keratinocyte growth media; KBM, keratinocyte basal media; INS, insulin; hTCEpi cells, human telomerized corneal epithelial cells. Scale bar: 5 μm. Adapted from Titone et al. (34).

Cunha and colleagues were the first to confirm a role for insulin in corneal metabolism (40). Studies in our laboratory have sought to further this work and define the mechanism by which insulin regulates cellular metabolism and growth in the corneal epithelium. To accomplish this, we first investigated the role of insulin in regulating cell cycle control in human corneal epithelial cells. After 48 h of growth factor withdrawal, corneal epithelial cells arrested in G0/G1. This arrest was partially restored following treatment with insulin for the final 24 h (11). In that same study, we determined the metabolic phenotype of corneal epithelial cells. We again found that insulin was able to partially restore mitochondrial respiration. This was not due to a shift in glycolysis however, but an increase in mitochondrial respiration (11). To further investigate these findings, we tested the effect of co-treatment with insulin when cells were cultured in basal conditions (no growth factors). Interestingly, we found that corneal epithelial cells undergo a metabolic fuel switch between 24 and 48 h of culture during growth factor withdrawal. In the first 24 h, metabolic activity is driven principally by mitochondrial respiration, whereas in the last 24 h, glycolysis is upregulated to account for a sudden decrease in respiration. In both conditions, insulin was able to maintain both respiration and glycolysis. Consistent with the measured drop in respiration at 48 h, fluorescent imaging showed that mitochondria were largely depolarized. Similar to its effect on respiration, co-treatment with insulin also blocked the loss of depolarization in these cells (Figure 4) (34). Together, these findings support that insulin promotes mitochondrial respiration in corneal epithelial cells by maintaining mitochondrial polarization.

Insulin is known to activate PI3K/Akt/mTOR signaling by first binding INSR or IGF-1R at the plasma membrane and then activating downstream cell survival pathways (46). In our laboratory, we showed that insulin regulates phosphorylation of Akt at ser473 in human corneal epithelial cells (11). Activation of this kinase cascade led to an increase in phosphorylation of GS3Kβ at the inhibitory residue, ser9. Since GS3Kβ is a key regulator of cell cycle control, mitochondrial function and apoptosis, and the autophagy inhibitor mTOR, phosphorylation of this residue leads to activation of mTOR and a block in autophagy (47, 48). Autophagy or macroautophagy is a cannibalistic mechanism used by cells to recycle damaged components and debris (49). Selective autophagy represents organelle-specific autophagy. Mitophagy, which is a key mitochondrial quality control mechanism, is the process whereby mitochondria are targeted to autophagosomes (50). In the presence of mitochondrial depolarization, as shown in our growth factor withdrawal model, PTEN-induced kinase 1 (PINK1) becomes stabilized in the mitochondria. PINK1 is a well-studied mitophagy marker that functions to recruit Parkin to the mitochondria. Parkin in turn ubiquitinates mitochondrial proteins, triggering recognition by the autophagosome for subsequent engulfment by the autophagolysosome. Similar to autophagy, insulin blocks all autophagic flux, including mitophagy, in corneal epithelial cells (34). In contrast to this, in breast cancer cells, IGF-1 has been shown to induce mitophagy through activation of the mitophagy receptor BNIP3 (51). Whether IGF-1 is able to activate macro- or selective autophagy in corneal epithelial cells is still unknown, but represents an exciting avenue for study.

## Insulin and the Diabetic Cornea

Several studies have described the effects of high glucose on corneal epithelial homeostasis through modulation of cell signaling, cell proliferation, and wound healing (7, 52–54). Clinically, these changes are manifested in the diabetic cornea in the form of superficial punctate keratitis, alterations in epithelial barrier function, recurrent epithelial erosions due to the presence of an abnormal basement membrane, persistent epithelial defects, and refractory wounds despite treatment (7, 54). In addition to cellular changes, the loss of corneal nerves drives a reduction in corneal sensitivity and this leads to epithelial thinning and reduced tear secretion. The latter of which underlies a cause of dry eye (53). The mechanisms leading to development of corneal complications are multifactorial and are due in part, to abnormal growth factor signaling and the accumulation of reactive oxygen species (53).

Due to the ability of insulin to promote proliferation in cell culture, topical insulin has been proposed as a treatment modality to promote corneal epithelial wound healing (Table 1). In their study, Zagon et al. used streptozotocin to induce diabetes in Sprague-Dawley rats (55). In this model, they made a 5 mm corneal wound, followed by treatment with topical insulin four times a day for 7 days. Compared to the vehicle control, topical insulin promoted epithelial resurfacing and an increase in proliferation of basal epithelial cells. Interestingly, treatment with topical insulin also restored corneal sensitivity to normal levels, suggesting that insulin also promoted corneal re-innervation.

**Table 1:** Published studies evaluating the role of IGF family ligands in the cornea.

**Molecular pathway**	**Wound repair**	**References**
Insulin	Promotes epithelial resurfacing and proliferation in rodent model.	(55)
	Enhances healing rates in patients with epithelial defects following vitrectomy.	(56)
	Dosed at 0.5 units QID was effective on healing epithelial defects post vitrectomy in diabetic patients without drug toxicity.	(57)
	Aides in wound repair through restoration of circadian rhythm in the corneal epithelium.	(58)
	Promotes healing in neurotrophic corneal ulcers.	(59)
IGF-1	Used with substance P accelerates corneal epithelial cell migration.	(60)
	Used with the substance P-derived peptide (FGML) increases epithelial migration.	(61)
	Used with Substance P promotes epithelial wound healing in an *in vivo* rabbit model.	(62)
	Used with substance P promotes epithelial attachment to fibronectin and Type VI collagen in a rabbit model.	(60)
	Identified the minimum sequence of substance P necessary for a synergistic wound healing effect.	(63)
	Used with substance P restores barrier function.	(64)
	Used with substance P increases wound healing and barrier function in capsaicin injected rat model.	(65)
	Used with FGLM-NH(2) promotes corneal epithelial wound healing in diabetic rats.	(66)
	C domain is the portion necessary for a synergistic wound healing effect with Substance P.	(67)
	Induces corneal epithelial cell migration and increases lamin-5 and β1-integrin expression.	(68)
	Used with FGLM-amide accelerates resurfacing of persistent epithelial defects.	(69)
	IGF-1 peptide sequence SSSR used with FGLM-amide accelerates wound healing.	(70)
	Upregulates IGF-1R expression in corneal epithelial limbal stem cells and drives differentiation during corneal regeneration.	(71)
	Used with substance P increases the rate of re-epithelialization in rabbits post PRK.	(72)
IGF-2	IGF-2R protein expression increases in corneal wound healing in order to regulate keratocyte differentiation to myofibroblasts.	(23)
	Increases along with its receptor in corneal injury. Aids in the proliferation of keratinocytes and synthesis of N-cadherin.	(73)

Bastion later reported on the results of a retrospective study evaluating the effects of topical insulin on healing epithelial defects in human diabetic patients who were subject to epithelial debridement while undergoing vitreoretinal surgery (56). Fifteen eyes of 14 patients (one patient had bilateral epithelial defects) were divided into one of three groups, diabetics who received topical insulin, diabetics who did not receive topical insulin, and non-diabetics who received topical insulin. In all cases where insulin was administered, re-epithelialization was accelerated compared to non-insulin controls. There were no cases of toxicity or adverse events reported. However, only five patients were evaluated per group. Fai et al. reported on the results of a much larger study (57). Over a 2 year period, all patients with epithelial defects following vitreoretinal surgery were recruited and randomized into one of four groups: 0.5, 1.0, or 2 units of insulin or a saline control. Patients receiving 0.5 units of topical insulin four times a day demonstrated the best efficacy, with clinical effects being lost at the highest dosage.

More recently, Wang and colleagues reported on a small case series of six human subjects with refractory corneal ulcers (59). Patients ranged in age from 2 to 73 years and all presented to clinic with non-healing neurotrophic corneal ulcers. Patients all had past ocular histories that included a battery of therapeutic treatments and surgical procedures that resulted in incomplete healing. In all six cases, one unit of insulin was administered at a frequency of 2–3 times a day and all patients resolved over a period of 7–25 days.

## Insulin and Circadian Rhythm

In rodent models, the proliferation of basal epithelial cells in the central cornea and epithelial regeneration following a wound are regulated in part by circadian rhythms (74–77). In addition, oscillations in the expression of core circadian genes, including *Clock* and *Bmal1*, have also been reported (74). While diabetes and other metabolic disorders are known to disrupt normal circadian rhythms, Song and colleagues investigated the impact of diabetes on circadian rhythms in the corneal epithelium using a Type 1 streptozotocin mouse model (58). After 2 weeks of disease, there was a significant change in key clock genes, a reduction in basal cell proliferation, and an increase in leukocyte infiltration in the limbal region. These effects were partially restored by the administration of systemic insulin. Since these studies were performed so early on following onset of diabetes, the circadian effects could be measured without the corresponding effects from loss of the subbasal nerve plexus. Thus, it is likely that alterations in the circadian rhythm reflect an early change in diabetes that contributes to disruption of normal homeostasis.

## Insulin and the Corneal Endothelium

The corneal endothelium is a single cell layer on the posterior aspect of the cornea that is flush with the aqueous humor. Function of the corneal endothelium is critical to maintain optimal hydration and stromal transparency. The role of IGF and insulin in the corneal endothelium has not been well-studied. In the bovine corneal endothelium, IGF-1R is ~25 times more highly expressed than INSR (78). This finding would explain why stimulation of bovine corneal endothelial monolayers with IGF-1 promoted DNA synthesis. While low levels of IGF-1 are able to not only stimulate DNA synthesis, they also induce an upregulation of the proto-oncogene c-fos. Unlike IGF-1, a high concentration of insulin is required to have a similar effect. This is likely due to the ability of insulin to activate IGF-1R at high levels.

Insulin has been shown to play a key role in the regulation of the Na/K-ATPase pump. The Na/K-ATPase pump functions to maintain water balance in the corneal stroma. In diabetes, endothelial cells are highly subject to damage during intraocular surgery and often display a certain degree of pleomorphism even in the absence of surgery (79). Corneal thickening in diabetes is thus due to improper pump function secondary to loss of corneal endothelial cells. In support of this, studies using an alloxan-induced diabetic rabbit model showed that a decrease in NA/K-ATPase activity was associated with an increase in corneal thickness and poor hydration control (80). Like glucose, insulin is present in the aqueous at a much lower concentration than serum. In alloxan-induced diabetes, levels of insulin in the aqueous are further depleted. To determine whether alterations in aqueous insulin levels in diabetes could account for the changes in pump function, Hatou et al. investigated the effect of insulin on Na/K-ATPase activity in mouse corneal endothelial cells *in vitro* (81). Importantly, they found that administration of insulin (0.01–10 μM) to an endothelial monolayer increased the activity of the Na/K-ATPase pump through activation of protein kinase C in a concentration dependent manner (82). It is important to note that these changes were transient in nature. Thus, a chronic insult from either no insulin in Type 1 diabetes or reduced signaling due to insulin resistance, such as that seen in Type 2 diabetes, may contribute to the pathophysiology of diabetes induced corneal endothelial damage.

## IGFs in Wound Healing and Repair

IGF-1 and -2 are homologous peptides that modulate cellular proliferation and differentiation throughout the body (83). Activation of their cognate receptors triggers autophosphorylation of their intracellular kinase domain, leading to downstream activation of the Janus kinase/signal transducers and activators of transcription (JAK/STAT), phosphoinositide 3-kinase (PI3K), and mitogen-activated protein kinase (MAPK) pathways (22, 84, 85). IGF-1 has been well-studied for its important role in cellular migration and proliferation in non-ocular tissues (86). In all three cell layers of the cornea, IGF has been shown to have critical regulatory functions that preserve homeostasis and promote wound repair. In the corneal epithelium, IGF-1 promotes proliferation. This occurs through activation of Hybrid-R and subsequent phosphorylation of Akt (19). IGF-1 was also shown by Lee and colleagues to induce corneal epithelial cell migration and increased expression of Lamin-5 and β1-integrin. These effects were mediated through the PI3K/AKT pathway (68).

Lastly, some data exists to support that IGF-1 also contributes to the differentiation of limbal stem cells into corneal epithelial cells. In their study, Trosan et al. found that after central epithelial debridement in the mouse cornea, IGF-1 and IGF-2 secretion is increased in corneal epithelial cells, while IGF-1R expression was increased in the limbus (71). Interestingly, the increase in IGF-1R expression in the limbus was driven by IGF-1 and promoted differentiation of limbal epithelial cells, evidenced by an upregulation of the cytokeratin K12. A subsequent study with a similar experimental design showed the same effect for IGF-2 (73).

## IGF and Substance P

Much of what is known regarding the function of IGF-1 in corneal epithelial wound healing is focused on the interactive role of IGF-1 with the neuropeptide, substance P (Table 1). In their *in vitro* studies, Nishida and colleagues showed that IGF-1 administered at a concentration of 10 ng/ml accelerated corneal epithelial cell migration across a wounded rabbit corneal stroma *ex vivo* when used in conjunction with 25 or 50 μg/ml substance P (60). Likewise, IGF-1 together with substance P, promoted corneal epithelial attachment to fibronectin and Type IV collagen. They further showed that this effect was not due to changes in ligand binding sites for IGF-1, but was mediated by interactions between substance P and the Tachykinin receptor, Nrk1 (17, 87). These findings were confirmed in a rabbit model subject to epithelial debridement using N-heptyl alcohol (62). Additional work by this group induced corneal neuropathy in a rat model by thermocoagulating the ophthalmic nerve that branches from the trigeminal ganglion (64). Using this model they showed that treatment of corneal epithelial wounds with substance P and IGF-1 improves barrier function in the corneal epithelium by promoting wound healing. Subsequent publications by this same group have further elaborated on these key findings and identified the specific amino acid sequences for both substance P and IGF-1 that are responsible for mediating these effects (63, 65–67, 69, 70, 72).

## IGF-1 in Tear Fluid

IGF-1 is present in tear fluid, although at very low levels (88, 89). In normal healthy conditions, the ratio of IGF-1 to IGFBP-3 is not sufficient to blunt the effects of IGF-1. However, in human diabetic tear fluid, the ratio of IGF-1 to IGFBP-3 is significantly reduced (89). Since IGF-1 binds IGFBP-3 with a greater affinity than IGF-1R, the shift in the IGF-1 to IGFBP-3 ratio is sufficient to sequester IGF-1 and inhibit the ability of IGF-1 to induce phosphorylation of IGF-1R or Hybrid-R (89). The inability of IGF-1 to promote proliferation in the diabetic corneal epithelium may contribute to delayed wound healing. IGF-1 and IGF-2 have both been shown to be upregulated during corneal wound healing. It is not clear whether either of these proteins are upregulated in the diabetic eye.

## IGF-1 and Stromal Keratocytes

The effects of IGF-1 are not restricted to the corneal epithelium, but also play an important regenerative role in the stroma. Corneal keratocytes, the primary cell type in the stroma, are essential for not only maintaining stromal structure but also form an interconnected, communication network within the cornea. It has been shown that IGFs play an important role in regulating formation of this network. Using the IGF-1R inhibitor, picropodophyllin (PPP), Berthaut found that the addition of IGF-1 in concert with PPP blocked both the number of tubules and interconnections formed by corneal fibroblasts cultured on Matrigel loaded with growth factors (90). IGF-1 is also critical in the process of keratocyte differentiation. During inflammation and wounding, stromal cells become activated and induce a differentiation program. Using a co-culture model, Ko et al. showed that both Simian virus 40-transformed human corneal epithelial cells (HCE) and primary cultured corneal fibroblasts secrete IGF-1 (91). Using siRNA knockdown of IGF-1 in HCEs, they further demonstrated that IGF-1 secreted by corneal epithelial cells induces N-cadherin expression, an adherens junction protein, in cultured corneal fibroblasts and that this was most likely regulated by the zinc finger protein, ZEB1. Unlike cancer cells, where the upregulation of N-cadherin is associated with downregulation of E-cadherin and the subsequent epithelial-mesenchymal transition, the increase in N-cadherin in corneal fibroblasts was not associated with changes in any other junctional proteins.

IGF-1 has also been shown to modulate the TGF-beta/SMAD signaling pathway, although the data is conflicting. Sarenac demonstrated that treatment of keratocytes with IGF-1 inhibited differentiation into myofibroblasts by attenuating TGF-beta signaling (92). They concluded that IGF-1 may be a viable therapeutic option to limit fibrosis during corneal wound healing. In contrast to this, Izumi found that IGF-1 stimulated proliferation of myofibroblasts during wound healing without first reverting cells back to their naïve state (93). This increased proliferation of myofibroblasts would further promote fibrosis. Taken together, these findings suggest that IGF-1 may induce differential effects on stromal cells depending on their differentiation status.

## IGF-2 in Stromal Keratocytes

IGF-2 has been shown to play a key role in development of the murine eye (94). The function of IGF-2 has also been investigated in postnatal corneal development. To accomplish this, Kane et al. used keratocytes harvested from bovine and rabbit corneas (95). They measured collagen production and secretion of IGF-2 and IGFBP-2. Striking differences were noted. Rabbit keratocytes, which were proliferative in culture, secreted both Type I collagen and IGF-2. In contrast, bovine keratocytes, secreted IGFBP-2 and not IGF-2. Culture of bovine keratocytes in conditioned media from rabbit keratocytes promoted proliferation and collagen deposition, suggesting that IGF-2 is important in collagen production. Using microarrays, gene expression was next evaluated in keratocytes obtained from mouse neonates and compared to adults. IGF-2 was the most abundant growth factor present. IGF-I and IGFBP-4 were also detected, but were expressed at much lower levels. Interestingly, prior work by this same group showed that IGF-2 was present in the bovine stroma, despite not being secreted by bovine keratocytes (96). They further demonstrated that IGF-2 was capable of inducing keratocyte proliferation without inducing myofibroblasts differentiation. While the source of IGF-2 in the bovine stroma was not determined, IGF-2 appears to be integral to early stromal development.

## IGF-1 and the Corneal Endothelium

There has been limited research done on the role of IGF-1 in the corneal endothelium. In embryonic corneal tissue, IGF-1 has been shown to promote DNA synthesis in endothelial cells (97, 98). Feldman and colleagues later used adult bovine corneal endothelium to test the effects of IGF-1 and insulin on DNA synthesis (78). Using BrdU labeling, they found that insulin and IGF-1 were both able to promote DNA synthesis. While both ligands were effective, insulin required much higher concentrations than IGF-1. This was due to the reduced affinity of insulin for IGF-1R. Another study evaluated the effects of IGF-1 on rabbit endothelial cells. In this study, Choi showed that IGF-1 promotes rabbit endothelial cell proliferation through the IRS-1 pathway (99). IGF-1 did not alter collagen production by these cells. Moreover, IGFBP-2 was produced by rabbit endothelial cells and functioned to sequester IGF-1. It is important to note however, that corneal endothelial cells do not undergo regeneration *in vivo* in cats, non-human primates, or humans. Thus, while IGF-1 promotes DNA synthesis and proliferation on corneal endothelial cells capable of mitosis, the effect of IGF-1 on the human corneal endothelium is relatively unknown.

## IGFBPs

IGF-1 and IGF-2 are secreted into the extracellular environment where they are bound to IGFBPs (100). Currently, there are six highly conserved IGFBPs. IGFBPs are found in serum and most extracellular fluids, including the aqueous humor and vitreous (101, 102). Due to the presence of the blood-retinal barrier, the origins of these binding proteins are thought to be tissues within the eye. Several groups have probed for the presence of IGFBP-3 in ocular tissues. Most of these studies have focused on the localization of IGFBP mRNAs. Arnold et al. were the first to investigate the distribution of the IGFBPs in the eye. Using northern blotting, they reported that mRNAs for IGFBP-2 and IGFBP-3 were present in bovine corneas, but their exact distribution was not specified (101).

In the developing chick embryo, the appearance of IGFBP-2 mRNA expression was found to be temporally and spatially controlled (103). Initially noted in the surface ectoderm at embryonic day 3.5 (E3.5), mRNA transcripts were detected in both the corneal epithelium and endothelium, as soon as the cornea began to develop into multilayers (E6). As development neared completion, IGFBP-2 transcripts were evident in all cells throughout the cornea. In a subsequent study, Burren and colleagues were able to confirm the presence of mRNA for IGFBP-2 in the cornea. In the rat eye, they found that IGFBP-2 localized to the basal layer of the corneal epithelium, keratocytes, and endothelium; however, they were unable to detect transcripts for any of the other binding proteins (104). More recently, expression of all six binding proteins was evaluated in a transgenic rat model that over-expressed the renin-2 gene (*REN-2*). The *REN-2* transgenic rat is a model for hypertension characterized by an alteration in the renin-angiotensin system that controls blood pressure (105). Type 1 diabetes was induced in this model using streptozotocin. The authors reported that transcripts for IGFBP-1, IGFBP-5, and IGFBP-6 were present in the cornea, with IGFBP-5 and -6 found to be expressed at the protein level throughout the cornea, including the corneal epithelium. Moreover, transcript levels for these two binding proteins were altered in diabetes, with IGFBP-5 levels increasing and IGFBP-6 levels decreasing.

## IGFBP-2 and Stromal Fibroblast Differentiation

IGFBP-2 has been found to have an important role in mediating differentiation of corneal fibroblasts (102). In this study, the authors demonstrated that human corneal keratocytes express high levels of IGFBP-2. In corneal fibroblasts cultured on plastic, increased levels of IGFBP-2 were associated with increased expression of aldehyde dehydrogenase (ALDH1A1) and keratocan, markers for quiescent keratocytes. In contrast, keratocytes cultured on plastic and treated with TGFβ transformed into myofibroblasts and expressed high levels of α-smooth muscle actin (α-SMA) and very low levels of IGFBP-2. This finding is consistent with the observation that TGFβ downregulates IGFBP-3 in dermal keratinocytes (106). Importantly, co-treatment of myofibroblasts with IGFBP-2 partially blocked this transformation through an increase in ALDH1A1, keratocan, and a partial loss of stress fibers, while siRNA knockdown of IGFBP-2 increased α-SMA. Collectively, these data indicate that IGFBP-2 may be a crucial protein that regulates the sequential transition of keratocytes into fibroblasts and myofibroblasts and provide further support of a critical role for the IGF system in corneal wound healing.

## IGFBP-2 and Pterygium

Recent data has shown a link between IGFBP-2 and human malignancies including prostate, ovarian, and colon cancer (107–109). Similarly, IGFBP-2 has been linked to pathological processes in the cornea. Pterygium, a non-cancerous conjunctival overgrowth onto the cornea, is known to express cellular markers that reflect increased proliferation and cellular invasion. Using cDNA microarrays, IGFBP-2 expression was increased in fibroblasts cultured from the pterygium body compared to conjunctival fibroblasts collected from normal tissue (110). In contrast, there were no differences in IGFBP-3 expression. This finding was confirmed at the protein level and suggests that aberrant IGFBP-2 expression may play a role in the development of pterygium.

## IGFBP-3

Unlike IGFBP-2, IGFBP-3 is highly regulated at the post-translational level by glycosylation and phosphorylation (111, 112). These post-translational modifications are hypothesized to regulate IGFBP-3s stability and function. In addition, IGFPB-3 is also regulated by synthesis rate and extensive proteolysis (113, 114). Many tissues produce IGFBP-3 locally, where it plays an important role in growth inhibition, including the corneal epithelium (10, 115, 116). IGFBP-3 has also been described as marker for senescence in cancer and in human fibroblasts (117, 118).

In dermal keratinocytes, IGFBP-3 has been shown to function as the main binding protein that interacts with IGF-1 to modulate proliferation (106). Consistent with this, altered expression of IGFBP-3 is associated with the development of psoriatic lesions (119). Izumi and colleagues used human corneal fibroblasts to show that treatment with TGFβ to induce α-SMA expression also upregulated IGF-1 and IGFBP-3 mRNA (93). As already discussed, the increase in IGF-1 has multiple effects including an increase in myofibroblast proliferation and stimulation of collagen production. Prolonged proliferation of myofibroblasts would contribute to excess fibrosis. IGFBP-3 on the other hand, modulates proliferation of myofibroblasts in an IGF-dependent manner. In the mouse cornea *in vivo*, following photorefractive keratectomy, IGFBP-3 was upregulated and expressed throughout the corneal stroma (93). IGFBP-3 has been shown to bind to certain extracellular matrix proteins and once bound, may alter its affinity for IGF-1. Thus, a potential temporal or spatial gradient in IGF-1 and IGFBP-3 may regulate the degree of fibrosis during corneal wound healing.

More recently, work by our laboratory has begun investigating the role of IGFBP-3 in mediating stress responses in the corneal epithelium. We have shown that IGFBP-3 is upregulated during growth factor withdrawal. This increase in IGFBP-3 is necessary to induce nuclear translocation of Hybrid-R (10). Once in the nucleus, IGF-1R and IGFBP-3 accumulate in the insoluble fraction (10). IGFPB-3 does harbor a nuclear localization sequence and has been shown to traffic to the nucleus in other cell lines and tissues (120). Most available data suggests that nuclear localization is important in regulating apoptosis. The function of nuclear IGFBP-3 in the cornea is unknown.

**Figure 5:**
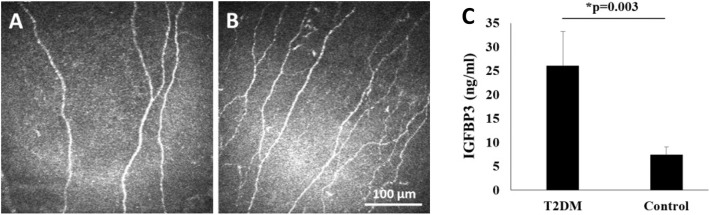
Human tear levels of IGFBP-3 correlate with loss of the subbasal nerve plexus in T2DM. **(A,B)** *In vivo* confocal microscopy of the corneal subbasal nerve plexus showing **(A)** fewer corneal nerve fibers and branches in T2DM; and **(B)** normal nerve morphology in the healthy, non-diabetic control. Scale bar: 100 μm. **(C)** Tear level of IGFBP-3 were increased in patients with T2DM compared to healthy controls (*P* = 0.003, *t*-test). Adapted from Stuard et al. (121).

While IGFBP-3 is necessary to induce trafficking of IGF-1R, loss of IGF-1R in turn downregulates IGFBP-3. Thus, IGFBP-3 and IGF-1R undergo mutual regulation to maintain homeostasis in the corneal epithelium (10). We have also found that IGFBP-3 secretion is increased in response to hypoxia (unpublished observations), and in response to hyperglycemia (89). In agreement with this latter finding, we have reported that human tear levels of IGFBP-3 are similarly increased in patients with diabetes (121). More importantly, the increase in tear levels of IGFBP-3 in Type 2 diabetes correlates with damage to the corneal subbasal nerve plexus (Figure 5) (121). What remains unknown is the size of IGFBP-3 present in human tear fluid and whether this is the full-length glycosylated protein or a smaller cleavage fragment.

## Summary and Future Directions

The IGF family is responsible for maintaining tissue homeostasis through the regulation of metabolic and/or mitogenic pathways at all cellular levels in the cornea. In addition to their canonical pathways, recent studies have led to the discovery of important intracellular functions in the corneal epithelium. This includes the nuclear translocation of Hybrid-R to the nuclei of human corneal epithelial cells in an IGF-1 independent manner and the ability of Hybrid-R to bind DNA and modulate gene expression. INSR and IGF-1R are also present in mitochondria where they likewise accumulate in the absence of IGF-1. The interaction of IGF-1R and INSR with VDAC1, a protein present in the outer mitochondrial membrane, suggests novel regulatory functions including the trafficking of molecules and ions, mitochondrial stability, and apoptosis. Further interrogation of these interactions may lead to the identification of critical new regulatory mechanism(s) that mediate mitochondrial function and quality control in the cornea and elsewhere in the body. These exciting new findings may also lead to the development of new therapeutic targets aimed at mitigating or preventing complications in patients with diabetes, where mitochondrial dysfunction is a central feature in the pathophysiology of disease.

One of the interesting characteristics that makes the corneal epithelium distinct from other epithelial tissues is the finding that insulin is not required for glucose uptake. Given the avascularity of the cornea, this is not altogether surprising. Insulin does have important regulatory roles in proliferation and cell growth in the corneal epithelium. These findings are not restricted to cell cultures *in vitro*, but extend to animal models and human studies. In diabetes, topical insulin does promote wound healing. The ability of insulin to restore circadian rhythm through *BMAL1* and *CLOCK* genes may facilitate re-epithelialization, as these rhythms may be disturbed in diabetes. One advantage of insulin compared to IGF-1 as a therapeutic option is that IGF-1 is a potent inducer of angiogenesis. While insulin does have angiogenic capabilities, it is not clear from the limited clinical data whether the development of neovascularization from topical use will develop.

In the last few years, several studies have focused on elucidating non-glucose transport functions for insulin. The most recent findings from our laboratory have demonstrated roles for insulin in the regulation of metabolic homeostasis through control of mitochondrial respiration, glycolysis, and autophagy. We have further shown that insulin regulates secretion of IGFBP-3, which in turn, mediates intracellular receptor trafficking. Interestingly, IGFBP-3 secretion is also mediated in part by IGF-1R. Taken together, these findings highlight the crosstalk that occurs between all the components of the IGF system. While the potential presence of proteases that further regulate IGF-1 bioavailability has not yet been investigated, it is clear that there is a delicate balance between members of the IGF-1 family that is critical for normal corneal development and tissue maintenance. Available evidence suggests that this balance is disrupted in diabetes and may contribute in part to recalcitrant wound healing. Moreover, since most of this work has been done in corneal epithelia, the role of insulin in keratocyte and endothelial health is relatively unknown and represents an important area of future study.

IGFBP-3 is a pleiotropic protein whose function is cell and context specific. Based on our prior studies, we hypothesize that IGFBP-3 functions as a major stress response protein in the corneal epithelium. In support of this view, tear levels of IGFBP-3 are increased in patients with diabetes and this increase correlates with loss of the subbasal nerve plexus. Much remains to be done to determine whether or not this discovery will lead to a novel diagnostic test that can be used to monitor patients with diabetes to determine potential risk for neuropathic or ocular complications. The advantages of using tears to monitor patients with diabetes include the ease and the relatively non-invasive nature of collection compared to phlebotomy. However, studies are needed to evaluate the impact of reflex tearing and dry eye on tear levels of IGFBP-3, and to determine its sensitivity and specificity compared to hemoglobin A1c.

In terms of wound healing, the major challenge facing clinicians today is fibrosis. While fibrosis may be disfiguring in skin, it is a leading cause of blindness in the cornea. In severe cases, fibrosis necessitates full thickness corneal grafts to restore vision. There is a growing body of evidence to indicate that IGF family members play an important role in fibrosis. This includes regulating the differentiation of keratocytes into fibroblasts and myofibroblasts and the induction of myofibroblast proliferation without reverting cells back to a fibroblastic phenotype. Much more data is needed to fully understand the contribution of this system to wound healing, the critical crosstalk amongst the differing cell layers in the cornea, and corneal development.

In conclusion, a new outline regarding the impact of IGF family members on the cornea is beginning to emerge. Huge gaps in knowledge persist, creating multiple new areas of much needed research. Future studies will not only allow us to fill in these gaps, but will also allow us to gain a greater appreciation for the function of insulin, IGF, related binding proteins, and proteases, in the normal cornea and in disease.

## Author Contributions

WS and RT wrote the manuscript. DR wrote and edited the manuscript.

### Conflict of Interest

The authors declare that the research was conducted in the absence of any commercial or financial relationships that could be construed as a potential conflict of interest.
